# A fluoroscopic view for midshaft clavicular fracture reduction and internal fixation: posteroanterior 25° skyline projection

**DOI:** 10.1186/s12893-022-01813-7

**Published:** 2022-10-29

**Authors:** Wentao Chen, Baojun Wang, Zhenyu Liu

**Affiliations:** Department of Orthopaedics, Beijing Friendship Hospital, Capital Medical University, 95 Yong’an Road, 100050 Xicheng, Beijing, P.R. China

**Keywords:** Midshaft, Clavicle, Fracture, Fluoroscopy, Posteroanterior

## Abstract

**Background:**

Open reduction and internal fixation have been frequently applied for displaced midshaft clavicular fracture. Plate and screw fixation of clavicular fractures could provide rigid fixation and rotational control. Proper implant positioning in surgical fixation is critical to prevent iatrogenic complications. Fluoroscopy plays an important role in the intraoperative evaluation of implants. This study aimed to introduce a new fluoroscopic projection to evaluate the positioning of plates and screws.

**Methods:**

Adult patients with a diagnosis of acute displaced midshaft clavicular fracture were included in this study. The slope angle of the midshaft clavicle was measured on sagittal reconstructions of preoperative computed tomography (CT) scans. The incidence of screw revision based on intraoperative standard posteroanterior (PA) and PA 25° cephalic skyline projections was compared. The interobserver agreement for the two projections was calculated.

**Results:**

Twenty-nine patients with midshaft clavicular fractures were enrolled from January 2020 to June 2021. The PA 25° skyline projection could clearly display the tangential line of the plate and inferior border of the clavicle. The slope angle on the superior surface of the midshaft clavicle was 26.0 ± 5.8° (range: 18.5–38.3°). The incidence of screw revision using the PA projection (72.4%) was significantly different from that using the PA 25° skyline projection (34.5%) (P < 0.05). The concordance of the screw revision rate based on the standard PA and PA 25° skyline projections was strong, with kappa coefficients of 0.680 (95% CI: 0.394–0.968) and 0.776 (95% CI: 0.537–0.998).

**Conclusion:**

The PA 25° skyline projection corresponds to the slope angle of the midshaft clavicle. It can provide more accurate information regarding the proper screw length and be applied as a routine method for intraoperative evaluation.

## Background

The incidence of midshaft fractures is approximately 80% of all clavicular fractures [1, 2]. Surgical intervention with plate fixation is recommended especially for the displaced midshaft clavicular fracture [3–5]. However, imperfect length and position of screws could result in screw tip protrusion, potentially leading to iatrogenic subclavian neurovascular bundle injury [6]. During the operation process, the fluoroscopy plays pivotal roles in fracture reduction and implants placement. Although the standard anteroposterior (AP) or posteroanterior (PA) projection was commonly applied as routine intraoperative method, it was not sufficient to determine the proper implants position due to irregular anatomical structure of clavicle. Various projections had been attempted to rule out evaluation of fracture reduction and implants position from different views to assure the fixation outcome and decrease the risk of iatrogenic complications. To date, no consensus had been made for optimal fluoroscopy.

Hereby the aim of this study was to introduce and compare a novel fluoroscopy projection with standard PA projection to clearly judge the position of plates and screws and signify its value in clinical practice.

## Methods

### Patient information

The patients from January 2020 to June 2021 met inclusion criteria were included in this study, while others were excluded (Table 1).

**Table 1:** Criteria of inclusion and exclusion

Inclusion	Exclusion
(1)age over 16 years	(1)pediatric fracture
(2)acute fracture	(2)pathological fracture
(3) displaced midshaft clavicular fracture (4) willing to accept open reduction and internal fixation	(3)open fracture
	(4)multiple fractures
	(5)nonacute fracture (over 2 weeks)
	(6)fractures of the medial and lateral thirds of the clavicle

### Surgical procedure

**Fig. 1:**
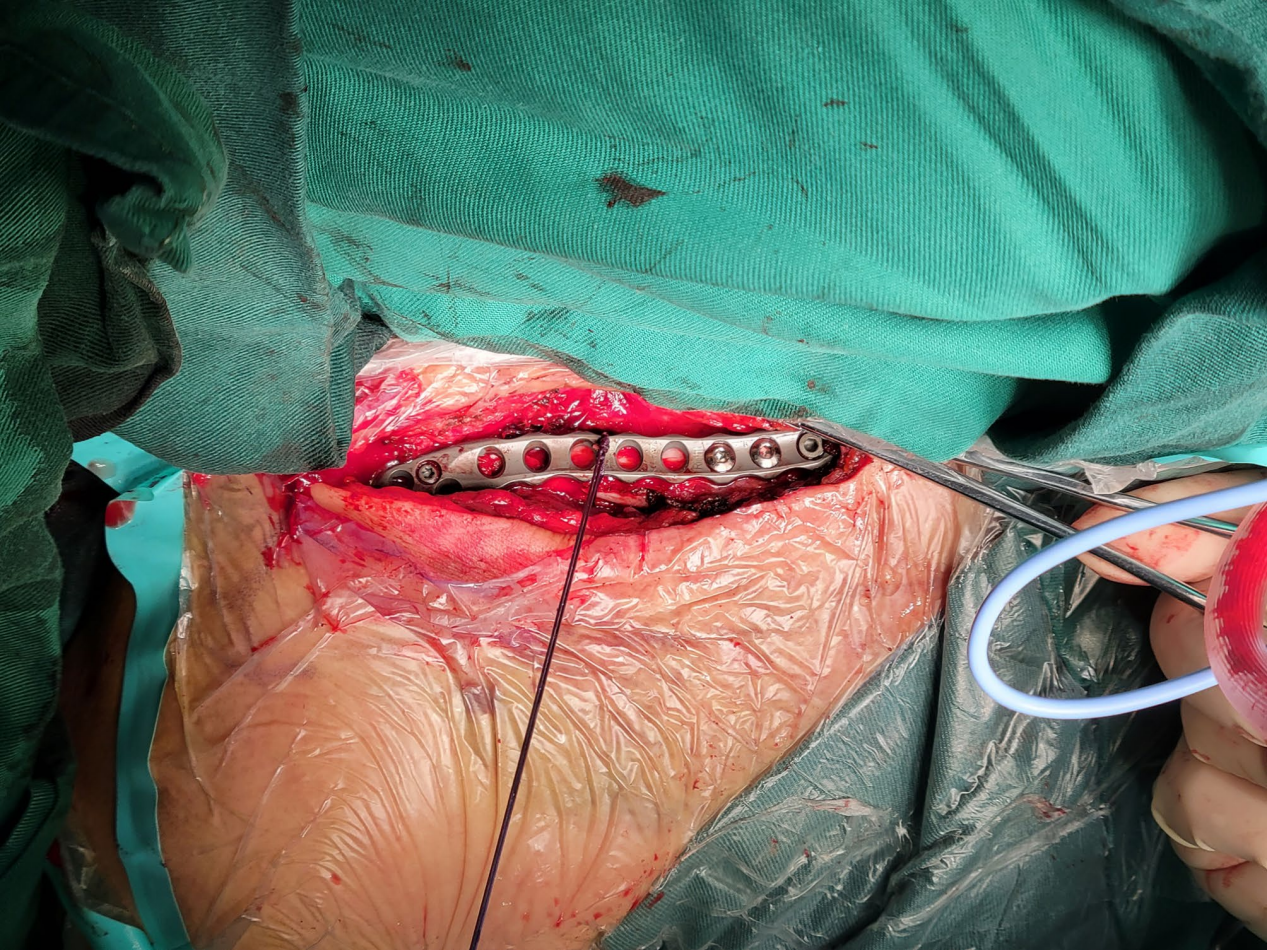
Intraoperative photograph of the open reduction and internal fixation of a displaced midshaft clavicular fracture with a superior precontoured plate and screws

All patients were lying supine on the radiolucent table with one small longitudinal pad placed between the scapulae. The ipsilateral arm was draped for intraoperative reduction. All operations were performed by 2 board-certified orthopedic surgeons with shoulder and elbow trauma fellowship training. An incision was made on the superior surface of the clavicle. After blunt dissection, reduction assisted with clamps, K-wires and Nice knots was applied to restore the anatomical structure of the clavicle. An anatomical precontoured plate (I.T.S. Gmbh, Lassnitzhohe, Austria) fitted to the S shape of the clavicle was placed on the superior surface of the clavicle (Fig. 1). Three screws with six cortices were applied on both the medial and lateral fragments to gain reliable fixation strength. Intraoperative fluoroscopy was used to monitor fracture reduction quality and guide the choice of plate and screws. Finally, the incision was closed after thorough irrigation and hemostasis.

### Radiographic evaluation

**Fig. 2:**
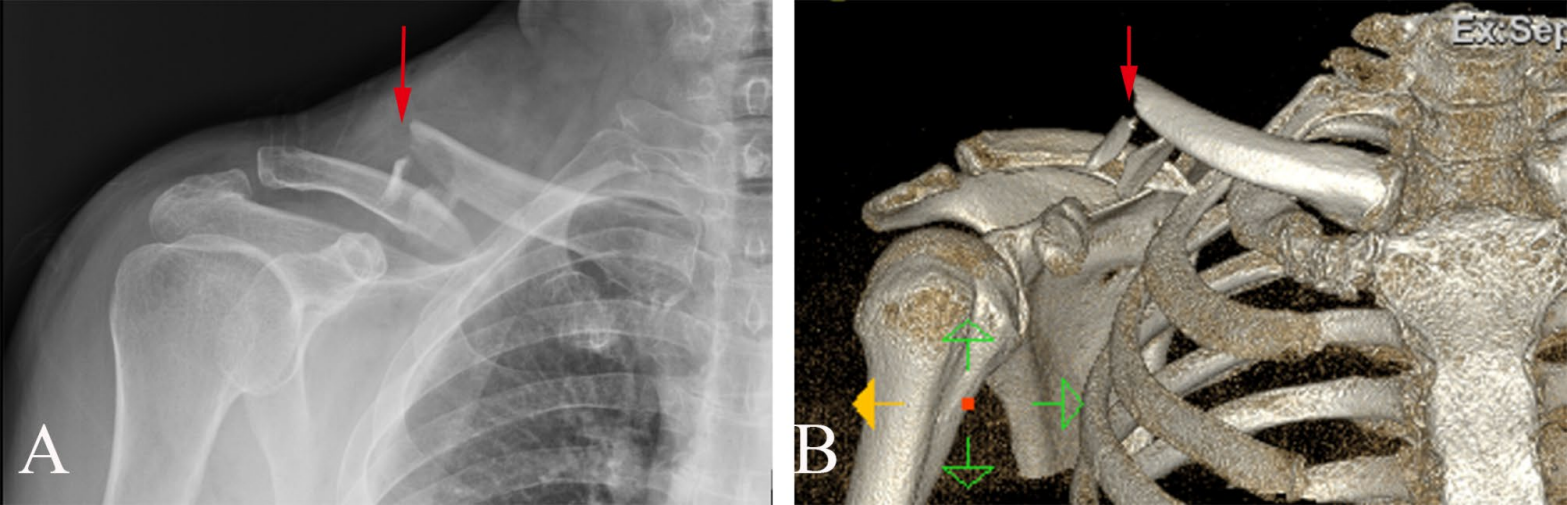
Preoperative AP radiograph and 3D CT reconstruction of a clavicular fracture A: AP radiograph. B: 3D CT reconstruction. The red arrow indicates the fracture site and comminuted fragments

**Fig. 3:**
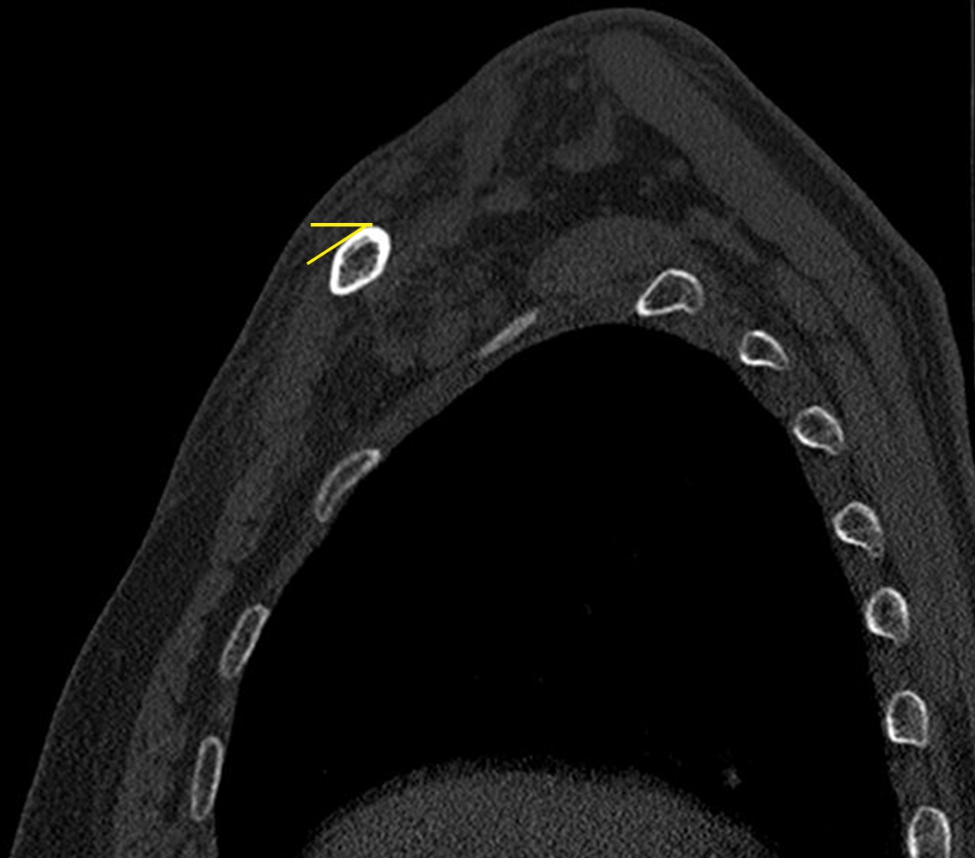
Illustrations of the slope angle measurement The yellow line represents the horizontal line and the superior slope

**Fig. 4:**
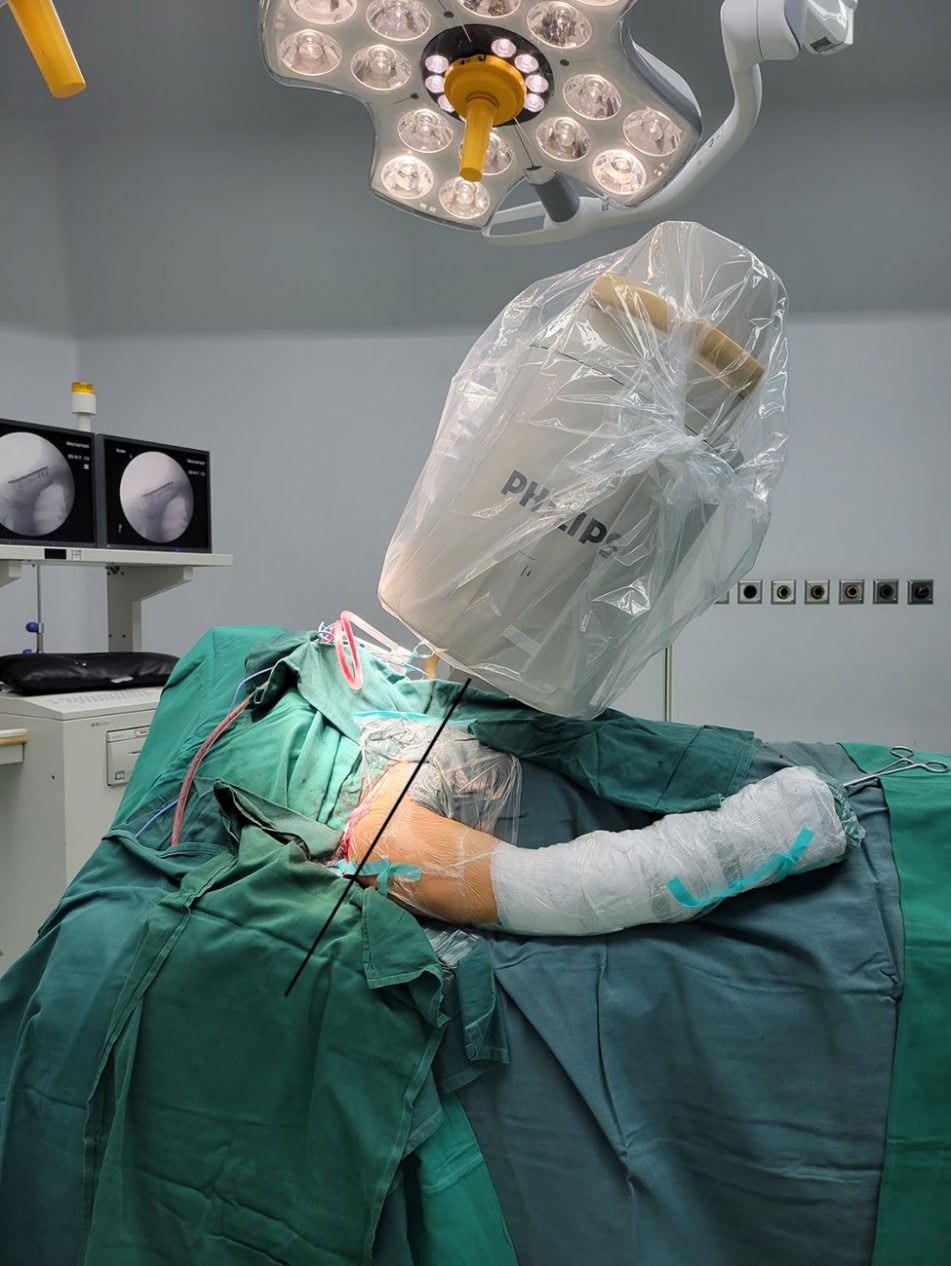
Clinical photograph: PA 25° cephalic skyline fluoroscopic projection of the clavicle with the patient in the supine position The X-ray beam (indicated with a black line) was centered on the middle point of the clavicle

Standard anteroposterior (AP) radiographs and 2D and 3D reconstructions of CT scans (GE Healthcare, Wauwatosa, WI, USA) were applied for preoperative diagnosis (Fig. 2). The slope angle was measured between the horizontal line and the slope line based on a sagittal sectional image of the midshaft clavicle on a workstation (Fig. 3). 2D intraoperative fluoroscopy was performed using a mobile C-arm unit (Philips Healthcare, Best, Netherlands). The base of the C-arm was located on the contralateral side of the patient, with no influence on the surgeons or surgical field. The X-ray tube was placed beneath the radiolucent operative table. The image intensifier with a sterile cover was the above patient, with access to the surgical field and no risk of contamination. The X-ray beam was focused on the middle point of the clavicle. Simulated posteroanterior (PA) fluoroscopy ranging from 0° to 30° of angulation in 5° increments was used (Fig. 4).

### Measurement of screws

The screw length was measured on intraoperative fluoroscopy images by one independent board-certified orthopedic surgeon with fellowship training in shoulder and elbow trauma. Unicortical screw without fixation of two cortices was considered short. Conversely, when the tip of the screw penetrated both cortices by more than two threads, it was considered long. The quality of fracture reduction and implant revision assessed on intraoperative fluoroscopy was documented, with a dichotomous result for screw revision, i.e., yes or no, by 2 independent board-certified orthopedic surgeons. The indication for revision was short or long screws. If there was any question about screw revision, final agreement was achieved through discussion.

### Statistical analysis

The incidence of screw revision based on intraoperative fluoroscopic imaging was compared and statistically analyzed using the chi-square test. The kappa coefficient was used to assess the interobserver agreement for the 2 fluoroscopic projections. P < 0.05 was considered statistically significant. The concordance rate was evaluated as follows: high, 0.81 < kappa < 1.00; strong, 0.61 < kappa < 0.80; moderate, 0.41 < kappa < 0.60; low, 0.21 < kappa < 0.40; and no concordance, kappa < 0.20. Statistical analysis was performed using SPSS Statistics (version 19, SPSS, Inc., Chicago, IL, USA).

## Results

From January 2020 to June 2021, 29 patients with unilateral displaced midshaft clavicular fractures were treated with open reduction and plate fixation. The mean age was 47.3 years (range: 16–76 years). The fractures were classified according to the Robinson classification system: all were type 2B (Table 2).

**Table 2:** Demographic data of 29 patients

Demographic data and detailed information of 29 patients	
Age (years)	47.3 ± 16.3
Sex (female:male)	8:21
Robinson classification	
2B1	25
2B2	4
Mechanism of injury	
Fall	10
Sports	9
Traffic accident	4
Other	6

**Fig. 5:**
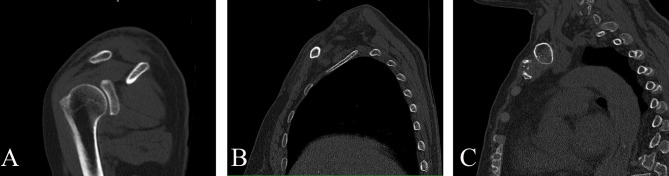
Sagittal sectional image of the lateral, middle and medial thirds of the clavicle A: Lateral third of the clavicle, flat B: Middle third of the clavicle, triangular C: Medial third of the clavicle, tubular

**Fig. 6:**
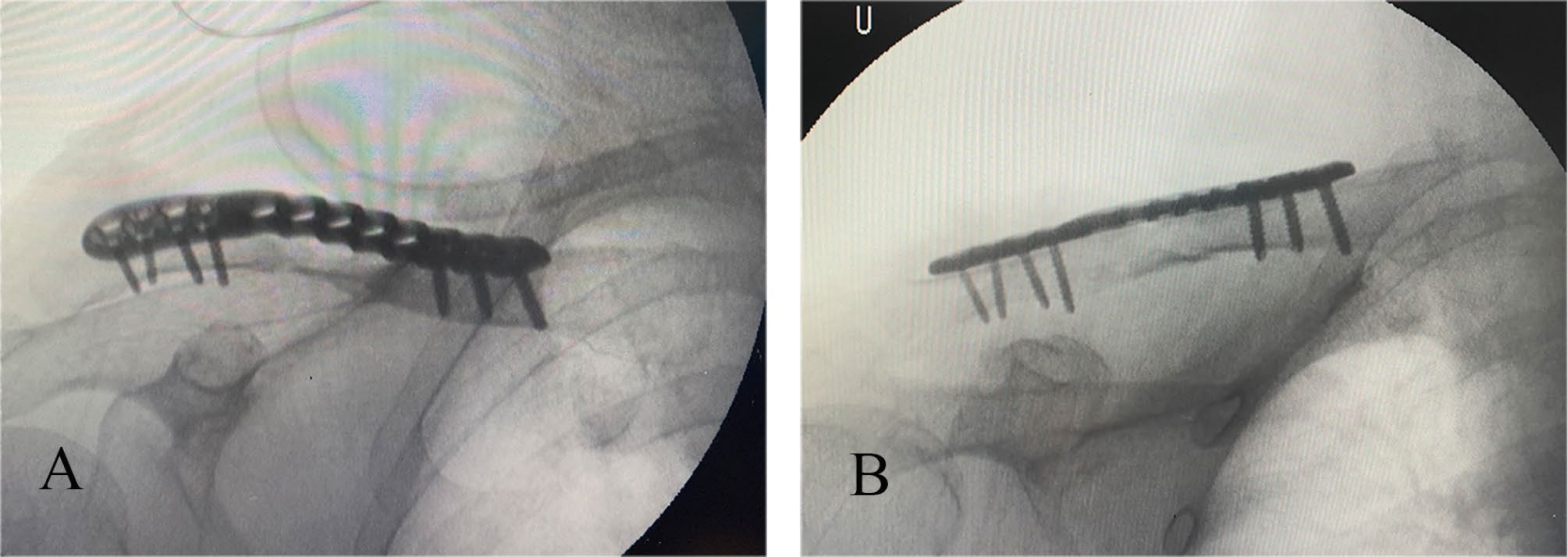
Different intraoperative fluoroscopic projections A: Standard PA projection B: PA 25° cephalic skyline projection

Sagittal 2D reconstructions revealed different anatomical geometries of the lateral, middle and medial thirds of the clavicle (Fig. 5). The slope angle on the superior surface of the middle third of the clavicle was 26.0 ± 5.8° (range: 18.5–38.3°). The different appearances of the fluoroscopic projections are shown in Fig. 6. The dichotomous results of screw revision using the two different projections judged by 2 observers are shown in Table 3. The incidence of screw revision using the PA projection (72.4%) was significantly different from that using the PA 25° skyline projection (34.5%) (P < 0.05). The interobserver agreement was good for the standard PA and PA 25° skyline projections. The concordance of the screw revision rate based on the standard PA and PA 25° skyline projections was strong, with kappa coefficients of 0.680 (95% CI: 0.394–0.968) and 0.776 (95% CI: 0.537–0.998).

**Table 3:** Screw revision using the standard PA and PA 25° skyline projections

Observer	Obs 1		Obs 2	
Projection method	Standard PA	PA 25° skyline	Standard PA	PA 25° skyline
Patient no.				
1	Y	N	Y	N
2	Y	N	Y	N
3	Y	N	Y	N
4	Y	N	Y	N
5	Y	Y	Y	Y
6	N	Y	N	Y
7	Y	Y	Y	Y
8	N	N	Y	N
9	Y	N	Y	N
10	Y	Y	Y	Y
11	N	N	N	N
12	N	N	N	N
13	N	N	N	N
14	N	N	N	N
15	Y	Y	Y	N
16	N	Y	Y	Y
17	Y	Y	Y	Y
18	Y	N	Y	N
19	Y	N	Y	N
20	Y	N	Y	Y
21	N	Y	Y	Y
22	Y	Y	Y	Y
23	N	N	N	N
24	Y	Y	Y	Y
25	N	Y	N	N
26	Y	N	Y	N
27	Y	N	Y	N
28	Y	N	N	N
29	Y	N	Y	N

Y means Yes: The length of screws was short or long; screw revision was required

N means No: The screw positions were appropriate for six-cortex fixation; no screw revision was required.

## Discussion

Generally, the majority of midshaft clavicular fractures can heal after nonoperative management. However, there is evidence that the nonunion rate after nonoperative management might be higher than previously reported, with nonunion rate in nonoperative patients reported to be 15-26% in recent studies [3, 4, 7]. Furthermore, plate fixation has demonstrated satisfactory results, with a high union rate [8]. There is an increasing trend in the incidence of clavicular fractures and application of internal fixation worldwide [9].The positioning of the plate on the superior surface or anteroinferior surface of the clavicle remains controversial. Superior clavicular plates have biomechanical advantages over anteroinferior plates in terms of fracture rigidity and failure bending load [10]. Meanwhile, superior plates also have a lower incidence of nonunion and a faster time to union [5]. Bicortical screws in superior plate fixation have been proved to be more rigid in fixation with biomechanical superiority, however they might increase the risk of adjacent neurovascular bundle injury [11, 12].These complications, including thoracic outlet syndrome, thrombosis of the subclavian vein and arterial pseudoaneurysm, had been described in several case reports [13–16]. Most were caused by prominent screw tip protruding from the inferior border of the clavicle into adjacent neurovascular bundle. Thus, directing screws away from the subclavian neurovascular bundle could prevent such iatrogenic complications. A previous study based on reconstructed 3D CT angiograms revealed that in the middle third of the clavicle, the minimum distance between the subclavian vessels and clavicle was 5 mm, with a safe zone for superior plate and screw placement [17]. Another MRI study revealed that the dangerous depth for subclavian neurovascular bundle injury was 17.3 (11.8–22.5)mm [18]. As previously described, the diameter of the middle third clavicle was 15 mm [6, 19], approximating the dangerous depth. Therefore, placement of screw with an appropriate length(maximum prominence less than 4 mm ) and position is helpful for preventing such iatrogenic injury [6].

Anatomically, the clavicle is a complex bone with two curves, anterior and posterior convex curves, and a unique sigmoid shape [20]. Due to the irregular structure, the clavicle is seldom located parallel to the X-ray film in different patient positions. Thus, it was difficult to acquire complete information regarding the clavicle by sole projection. Many factors could influence the image findings of claviclular fractures, such as patient position(upright vs. supine), the projection angle and chest rotation.

To our knowledge, a variety of fluoroscopy techniques were reported to evaluate clavicle fracture and internal fixation. The AP view, a 15° or 45° cephalic tilt view [21], the abduction lordotic view [22] and the apical oblique view [23] were good preoperative techniques to assess fracture displacement and shortening. Some techniques required manipulation of the injured arm or special patient position, which was not feasible in operation. Simultaneously, there was inability for these techniques to judge fracture realignment and gauge accurate screw length.

In addition, CT scans and 3D reconstruction image could provide more accurate information of fracture reduction, plate position and screw length. However, intraoperative CT is not routinely available in most scenarios. 2D fluoroscopy using a mobile C-arm is a widely used and convenient method for the intraoperative evaluation of implant characteristics. Different projection angles have been described for intraoperative fluoroscopy in the assessment of fracture reduction and plate placement, ranging from 45°cephalic to 45°caudal [24, 25]. However, the exact angle for different surgical patient position(supine or beach chair) has still not been defined. No consensus on the standardized fluoroscopy projection for the intraoperative evaluation has been built yet.

The sagittal cross-section of the clavicle has been reported to be irregularly shaped, changing from flat laterally to tubular centrally to triangular medially [19, 26]. In contrast to previous findings, our CT scans revealed the sagittal cross-section of the middle third of the clavicle was triangular. The superior plate was usually placed on the slope of the midshaft clavicle, which was on the tension side of the bone. The slope angle of the midshaft clavicle was 26.0 ± 5.8° based on CT measurements. Although accurate length of the screws could be gauged by surgical experience and measuring device, bicortical screw might be misjudged as unicortical through conventional AP view. The incorrect information could mislead surgeons to replace the indicated short screws with longer ones with a protruding tip, thereby increasing the risk of adjacent subclavian neurovascular bundle injury. A proper screw placement is usually achieved by surgical experience, measuring device and eventually confirmed by intraoperative fluoroscopy.

In contrast, the PA 25° skyline projection, which corresponded to the slope angle, could clearly display the tangential line of the plate and inferior border of the clavicle, allowing the surgeons to judge the length of the screws and determine whether revision was required to avoid excessive screw protrusion. In this study, the PA 25° cephalic skyline projection decreased the rate of intraoperative revision for screw malpositioning to 34.5%.

Therefore, we recommend the PA 25° skyline projection for assessing midshaft clavicular fracture reduction and selecting suitable superiorly based plate and screws. This projection demonstrated better implants characteristics, with no manipulation of the injured upper extremity. With direct visualization of fracture reduction and superior plate placement, the PA 25° skyline projection improved the confidence of the surgeon in screw placement with little radiation exposure.

There are still several limitations to this study. First, although with adequate statistical power for the chi-square test and kappa coefficients, only 29 patients were included in this study. As reported, there are intra and inter individuals morphology variations of clavicle. Thus, results from a larger sample might be more robust. Second, the screw revision rate in this study might be higher than others. Small sample size and variable technical aspects between surgeons might contribute to high screw revision rate. Improvement of surgical techniques will be beneficial to decrease this rate in larger sample study. Another limitation is the necessity of intraoperative and postoperative CT evaluations. While it could provide more accurate information regarding screw position and length, the radiation dose and economic expenditure of CT are higher than conventional fluoroscopy. CT scan is commonly not available for intraoperative and postoperative radiological evaluation.

## Conclusion

The PA 25° skyline projection corresponds to the slope angle of the midshaft of the clavicle. It can provide more accurate information regarding the proper screw length and can be applied as a routine method for intraoperative evaluation.

## Data Availability

The datasets supporting the conclusions of this article are available in the figshare repository, 10.6084/m9.figshare.19126913.

## List of abbreviations

AP: anteroposterior
PA: posteroanterior
